# Identification of VOCs in essential oils extracted using ultrasound- and microwave-assisted methods from sweet cherry flower

**DOI:** 10.1038/s41598-020-80891-0

**Published:** 2021-01-13

**Authors:** Huimin Zhang, Hongguang Yan, Quan Li, Hui Lin, Xiaopeng Wen

**Affiliations:** 1 Key Laboratory of Plant Resource Conservation and Germplasm Innovation in Mountainous Region (Ministry of Education), Institute of Agro-bioengineering, Guizhou University, Guiyang, 550025 People’s Republic of China; 2 Institute for Forest Resources & Environment of Guizhou/College of Forestry, Guizhou University, Guiyang, 550025 People’s Republic of China; 3 Kaili University, Kaili, 556011 Guizhou China; 4 College of Chemistry and Materials, Ningde Normal University, Ningde, 352100 China

**Keywords:** Plant sciences, Chemistry

## Abstract

The floral fragrance of plants is an important indicator in their evaluation. The aroma of sweet cherry flowers is mainly derived from their essential oil. In this study, based on the results of a single-factor experiment, a Box–Behnken design was adopted for ultrasound- and microwave-assisted extraction of essential oil from sweet cherry flowers of the Brooks cultivar. With the objective of extracting the maximum essential oil yield (w/w), the optimal extraction process conditions were a liquid–solid ratio of 52 mL g^−1^, an extraction time of 27 min, and a microwave power of 435 W. The essential oil yield was 1.23%, which was close to the theoretical prediction. The volatile organic compounds (VOCs) of the sweet cherry flowers of four cultivars (Brooks, Black Pearl, Tieton and Summit) were identified via headspace solid phase microextraction (SPME) and gas chromatography–mass spectrometry (GC–MS). The results showed that a total of 155 VOCs were identified and classified in the essential oil from sweet cherry flowers of four cultivars, 65 of which were shared among the cultivars. The highest contents of VOCs were aldehydes, alcohols, ketones and esters. Ethanol, linalool, lilac alcohol, acetaldehyde, (*E*)-2-hexenal, benzaldehyde and dimethyl sulfide were the major volatiles, which were mainly responsible for the characteristic aroma of sweet cherry flowers. It was concluded that the VOCs of sweet cherry flowers were qualitatively similar; however, relative content differences were observed in the four cultivars. This study provides a theoretical basis for the metabolism and regulation of the VOCs of sweet cherry flowers.

## Introduction

Volatile organic compounds (VOCs) represent an important part of the plant metabolome and attract agronomic and biological interest due to their contribution to fruit aroma and flavor and, therefore, to fruit quality. Analysis of the composition of VOCs in plant flowers is a prerequisite for the application of their extracts^1,2^. The extraction/isolation techniques of VOCs include simultaneous distillation and solvent extraction (SDE), supercritical fluid extraction (SFE), hydrodistillation (HD), microwave-assisted hydrodistillation (MAHD), cold-press (CP), headspace (HS) extraction, and solid phase microextraction (SPME)^3–6^. Gas chromatography–mass spectrometry (GC–MS) is commonly employed to detect VOCs. Conventional extraction methods (such as SDE, HD, etc.) have problems such as a low extraction rate and weak extract activity, which restrict the development and application of plant extracts. Therefore, microwave, oscillation, and ultrasonication extraction methods have also been proposed. The purpose of using the combined ultrasound- and microwave-assisted extraction (UMAE) technique for the extraction is to alternative conventional extraction techniques, because UMAE is inexpensive, simple, rapid, green and efficient. Generally, UMAE will not affect the quality of essential oil extraction and the extraction yield was significantly increased via the UMAE method instead of conventional extraction methods^7–10^. e.g. UMAE was usually employed to extract essential oils, total flavonoids, dihydroquercetin etc. from seed, flower, bark, xylem of plant^11–13^. As a viable extraction technique, this method combined with GC/MS or UPLC/MS can be used as a routine analysis strategy for essential oil in plant. Due to the rapid development of analytical instruments and sample preparation techniques, floral fragrance research has gradually intensified. By the detection and analysis of HS-SPME/GC–MS, the VOCs of many common plant flowers have been initially identified^14^.

Many plants and flowers in nature emit aromas, and many aroma components are mixed to form a floral fragrance; different aroma components and their content differences provide their unique characteristics^15^. Floral fragrance is one of the important traits that are considered in evaluating ornamental plants. The fragrances of plant flowers are derived from various volatiles released from petals or flowers. Studies have shown that aroma is derived from the secondary metabolites of flowers, and major components are volatile small molecule compounds, such as terpenes, phenylpropionic acids and fatty acid derivatives^16,17^. During the flowering process, the types, contents and proportions of aroma components have a strong influence in attracting pollinators to forage. By volatilization, the smell of some flowers not only generates a signal to stimulate the immune response to self-heal but also has an important role in plant resistance to various stresses^18^. The VOCs of many plants possess obvious aromatic effects and medicinal value. The research focus on floral fragrance gives attention to the aroma of flowers with metabolomics and genomics^19,20^ and focuses on the relationship between aroma components and regulatory mechanisms^21,22^.

Sweet cherry (*Prunus avium* L.), which is also known as large cherry, is a fleshy stone fruit that belongs to the genus *Prunus*, and it is grown in specific regions with temperate climates^23^. The color of sweet cherry fruit is bright, and the fruit is sweet, delicious, and rich in nutrients^24^. In southern China, the introduction and cultivation of sweet cherries has experienced problems, such as a low rate of fruit set, serious fruit drop, unstable yield and fruit malformation, which have seriously affected the development of the sweet cherry industry in the region. Some scholars have explained the effect of temperature on the flowering and fruiting of sweet cherry introduced in the southern region from the aspects of cold demand, flower bud differentiation process and rate, embryo development, and quality and fruit setting^25^. Lech et al.^26^ analyzed the flowering of several sweet cherry cultivars in the climatic conditions of southern Poland. In addition, Legua et al.^27^ investigated the bioactive and volatile compounds in sweet cherry fruits. However, related research on the VOCs of sweet cherry flowers is lacking. The aroma of sweet cherry flowers is mainly derived from its essential oil. In this experiment, sweet cherry flowers from four cultivars were utilized as the research object; their essential oils were extracted, which provide a reference for the utilization of the essential oil of sweet cherry flowers. The identification of their VOCs will provide a scientific basis for elucidating the floral fragrance formation mechanism, metabolism and regulation.

## Materials and methods

### Plant material

The samples (blooming flowers were gathered in early April) of the sweet cherry cultivars Brooks, Black Pearl, Tieton and Summit were collected in 2020 from the Baiyi fruit tree experimental base of Guiyang Wudang at the Guizhou Institute of Fruit Tree Science (27°03′3.89″N and 106°25′47.23″E). The trees were 5 years old, and they were grafted onto “Gisela 6” root stock and planted under rain-shelter conditions in March 2015. All trees were managed with the same practical techniques. Identification of plant material was initially performed using morphological features and then confirmed by Prof. Xiang Wang at the Kaili University. A voucher specimen (No. 1033) has been deposited in the herbarium of our laboratory. The samples (well-developed flowers from the base of the shoots) from the same location were harvested from each tree. After they were washed with distilled water, the fresh samples were immediately loaded into a sampling bottle with a 50 mL-vial volume, in which the flower sample was placed. The fresh samples were tested two hours after they were transported to a laboratory.

### Extraction method

The 5.000 g flower sample of the Brooks sweet cherry cultivar was placed in a special flask for UMAE. Methanol:chloroform:ddH_2_O (5:2:2, v/v/v) mixed solution was added, and the flask was placed in the UMAE apparatus. The microwave extraction power was adjusted; the ultrasonic power was set to 50 W; the extraction time was set; the filter was vacuumed the filter residue was discarded; and then the collected extracting solution was poured into a rotary evaporator. The next step was to retrieve solvents by reduced pressure distillation, and the extracts were obtained at the same time.

**Figure 1:**
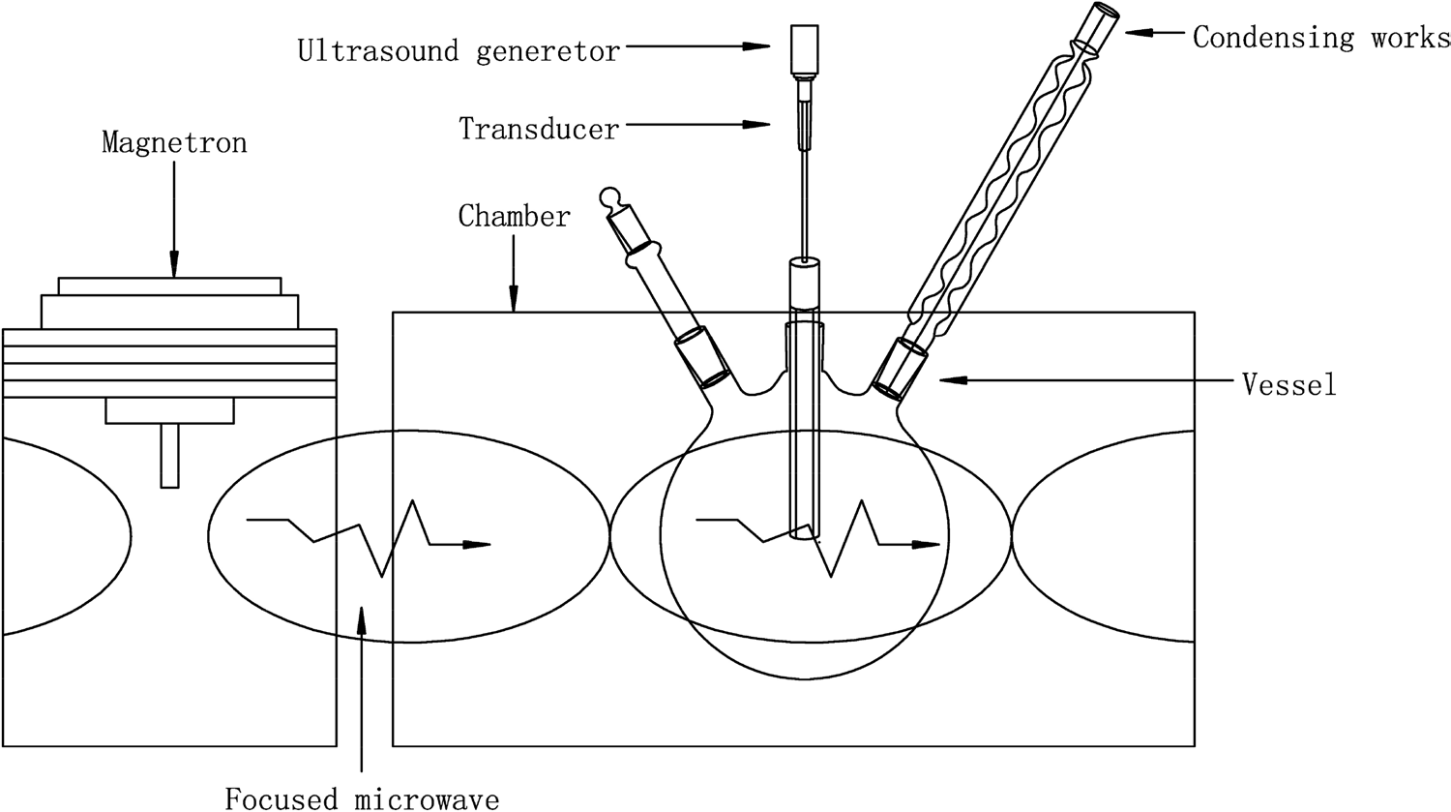
Schematic diagram of simultaneous ultrasound- and microwave-assisted extracting apparatus.

A simultaneous UMAE apparatus (XO-SM, Nanjing Xianou Instrument Manufacturing Co., Ltd., China) shown in Fig. 1 was applied for sample extraction.

The parameter of UMAE show in Table 1; Rotary Evaporator; model R-201D, produced by Shanghai Yike Instrument Co., Ltd. (Shanghai, China); and 7890B-5977B GC–MS, Agilent Technologies Co. Ltd. (California, USA). Methanol (CAS: 67-56-1) and chloroform (CAS: 67-66-3) were purchased from Tianjin Sayfo Technology Co., Ltd. (Tianjin, China).

**Table 1:** Parameter of ultrasound- and microwave-assisted extracting apparatus.

Model	Ultrasonic power	Ultrasonic frequency	Microwave power	Microwave frequency	Capacity	Ultrasonic probe diameter
XO-SM	0–900 W	25 kHz	0–700 W	2450 MHz	0.5–500 mL	Φ 6 mm

### Single-factor experiment

The purpose of this single-factor experiment was to investigate the effects of three factors (liquid–solid ratio, extraction time, and microwave power) on the yield (w/w) of sweet cherry flower essential oil (Table 2).

**Table 2:** Factors and levels in the single-factor experiment.

Factors	Level					
	1	2	3	4	5	6
A Liquid–solid ratio/mL g^−1^	10	20	30	40	50	60
B Extraction time/min	5	10	15	20	25	30
C Microwave power/W	100	200	300	400	500	600

### Response surface methodology experiments

For the response surface methodology and experimental design scheme, three factors were selected as independent variables, namely, the liquid–solid ratio, extraction time and microwave power, and the yield of sweet cherry flower essential oil was the response value.

### HS-SPME/GC–MS conditions

According to the best process parameters obtained by optimizing the extraction of Brooks flower essential oil, Black Pearl, Tieton and Summit flower essential oils were extracted. After sampling, the 1 mL samples were immediately submitted to the HS-SPME/GC–MS system. Volatiles were isolated by SPME (50 °C), which included oscillation for 15 min and extraction for 30 min (250 RPM) using the CTC triad automatic sampler (Extractor head: 50/30 μm DVB/CAR on PDMS). Gas chromatography was performed on a DBwax (30 m × 0.25 mm × 0.25 µm) to separate the volatile compounds at a constant flow of 1 mL min^−1^ helium. The injection temperature was 260 °C. The temperatures of the column and ion source were 40 °C and 230 °C, respectively. The temperature-rise program was followed by an initial temperature of 5 °C for 5 min, 20 °C min^−1^ with a maximum rate of 250 °C and then held constant for 2.5 min. Mass spectrometry (EI^+^, 70 eV) was determined by a full-scan method with a range from 200 to 400 (m z^−1^). The classification was referred from NIST 2014^28–30^.

### Statistical analyses

Statistical analysis was performed using the SPSS Statistics Version 21.0 software (Chicago, IL, USA). Duncan’s multiple comparisons test (p < 0.05) was performed to assess the statistically significant differences between the mean values (means) of three replications. Origin 9.1 (Origin Lab, Northampton, MA, USA) was employed to construct the graphs.

## Results and discussion

### Selection of factors and their levels by single-factor analysis

**Figure 2:**
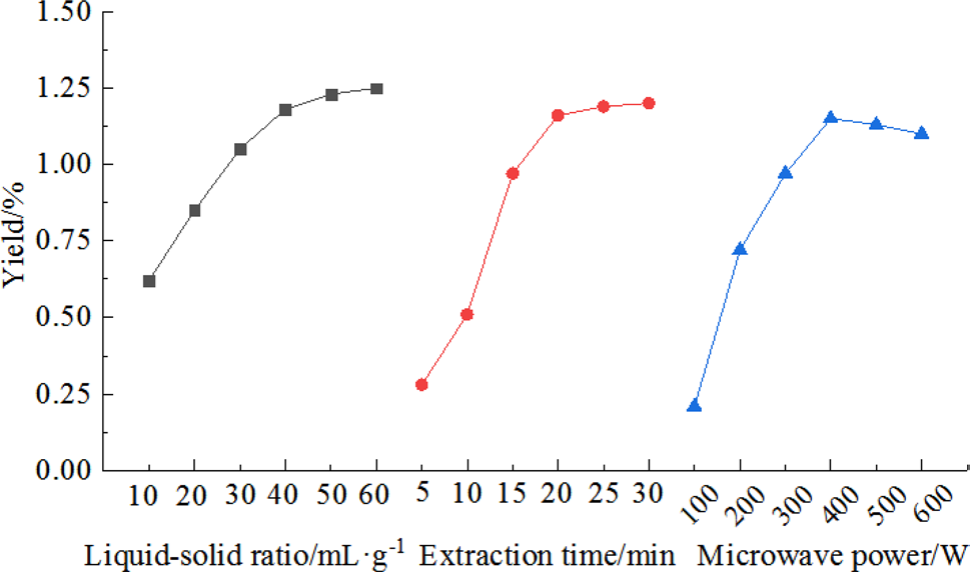
Effect of the liquid–solid ratio, extraction time and microwave power on the yield of sweet cherry flower essential oil.

With an extraction time of 20 min and a microwave power of 400 W, the influence of the liquid–solid ratio on yield was investigated; the results are shown in Fig. 2. As the amount of solvent increases, the yield of sweet cherry flower essential oil increases and then becomes stable. When the liquid–solid ratio is 60 mL g^−1^, the yield approaches the maximum, perhaps because as the amount of solvent increases, the contact area between the solvent and the raw material increases, and the essential oil can be fully dissolved. However, when the amount of solvent is too high, the solvent absorbs more microwave energy, and the absorption of microwave energy by the raw material decreases, which causes a decrease in the ability of the microwave to damage raw material cells, and the yield of essential oils only slightly changes^31^. After comprehensive consideration, a liquid–solid ratio of 40 mL g^−1^ is selected.

With a liquid–solid ratio of 40 mL g^−1^ and microwave power of 400 W, the effect of extraction time on the yield of essential oils was investigated. It can be seen from Fig. 2 that with an extension of extraction time, the yield increases and then gradually becomes stable with minimal change after 20 min. In a certain period, with an extended microwave treatment time, the raw material absorbs more microwave energy; the cell rupture is more sufficient; and the essential oil dissolves more. Thus, the yield continues to rise. However, as the microwave treatment time continues to increase, the essential oil inside the raw material is completely extracted, and further increases in the extraction time have a minimal effect on the yield^32^. Therefore, 20 min is an appropriate extraction time.

With a liquid–solid ratio of 40 mL g^−1^ and an extraction time of 20 min, the influence of microwave power on yield was investigated. The results are shown in Fig. 2. As the microwave power increases, the yield gradually increases and reaches a maximum at 400 W. At a power greater than 400 W, the yield decreases slightly. A microwave extracts essential oils by desorption. With an increase in microwave power, the heating rate increases; the cell wall quickly ruptures; and a large amount of essential oils dissolves. However, when the microwave power is too high, the heat-sensitive compounds in the essential oil will oxidize and decompose, which causes a decrease in yield. The experimental results of this study are consistent with the viewpoint of Routray and Valérie^33^. Usually, the microwave extraction yield will increase with the temperature, and after a certain optimum temperature is reached, further heating will not increase the yield. This result may be due to the increase in molecular migration and solute dissolution rate during the heating process. However, if the temperature is too high, the energy consumption will increase, and the extraction yield will not increase significantly. Therefore, the optimum microwave power is 400 W.

### Model fitting and effect of UMAE factors on yield

Based on the results of a single-factor experiment, three factors were selected as independent variables: the liquid–solid ratio (A), extraction time (B), and microwave power (C). The essential oil yield (Y) was the response value. The factors and levels are shown in Table 3. The Box–Behnken design and results are shown in Table 4. The binary polynomial regression model equation fitted by the software is:

1 $$\begin{aligned} {\text{Y }} & = 1.23 + 0.023{\text{A }} + 0.012{\text{B }} + 0.045{\text{C }} + 0.017{\text{AB }} \hfill \\ & \quad - \, (2.500{\text{E}} - 003){\text{AC }} + (2.500{\text{E}} - 003){\text{BC }} - 0.068{\text{A}}^{{2}} - 0.023{\text{B}}^{{2}} - 0.063{\text{C}}^{{2}} \hfill \\ \end{aligned}$$

**Table 3:** Factors and levels in the response surface design.

Factors	Level		
	− 1	0	1
A Liquid–solid ratio/mL g^−1^	40	50	60
B Extraction time/min	20	25	30
C Microwave power/W	300	400	500

**Table 4:** Box–Behnken experimental design and results.

No	A Liquid–solid ratio	B Extraction time	C Microwave power	Y Yield/% (w/w)	No	A Liquid–solid ratio	B Extraction time	C Microwave power	Y Yield/% (w/w)
1	60	25	500	1.20	10	50	25	400	1.23
2	60	25	300	1.08	11	50	30	300	1.13
3	40	20	400	1.14	12	50	20	500	1.16
4	60	30	400	1.18	13	50	25	400	1.23
5	50	20	300	1.11	14	60	20	400	1.12
6	50	25	400	1.25	15	50	25	400	1.22
7	40	25	300	1.00	16	40	25	500	1.13
8	50	25	400	1.24	17	50	30	500	1.19
9	40	30	400	1.13					

A variance analysis was performed using the regression model equation, and a significance analysis was performed using the model coefficients. As shown in the Table 5 model variance analysis results, the Model P-value of 0.0025 implies that the model is significant. The probability of this large “Model F-Value” is due to noise. Values of “Prob > F” less than 0.05 indicate that the model terms are significant. R^2^ = 0.9318 and Adj R^2^ = 0.8442 are near 1, which means that a 93.18% variation in the yield (w/w) of sweet cherry flower essential oil can be explained by the model. The quadratic polynomial regression model employed in the experiment shows a high degree of significance, which indicates that the experimental data fit the regression mathematical model and can better predict the actual values of various indicators^34^.

**Table 5:** Analysis of variance for the regression model.

Source	Sum of squares	Degrees of freedom of variation	Mean square	F value	Prob > F
Model	0.065	9	7.25E−03	10.63	0.0025
A-Liquid–solid ratio	4.05E−03	1	4.05E−03	5.94	0.0449
B-Extraction time	1.25E−03	1	1.25E−03	1.83	0.2177
C-Microwave power	0.016	1	0.016	23.77	0.0018
AB	1.23E−03	1	1.23E−03	1.8	0.2219
AC	2.50E−05	1	2.50E−05	0.037	0.8535
BC	2.50E−05	1	2.50E−05	0.037	0.8535
A^2^	0.02	1	0.02	28.78	0.001
B^2^	2.28E−03	1	2.28E−03	3.34	0.1103
C^2^	0.017	1	0.017	24.72	0.0016
Residual	4.77E−03	7	6.81E−04		
Lack of fit	4.25E−03	3	1.42E−03	10.9	0.0215
Pure error	5.20E−04	4	1.30E−04		
Cor toal	0.07	16			
R^2^	0.9318				
Adj R^2^	0.8442				
Pred R^2^	0.0166				
C.V.%	2.25				

Values of “Prob > F” less than 0.05 indicate that the model terms are significant, and values greater than 0.10 indicate that the model terms are not significant.

**Figure 3:**
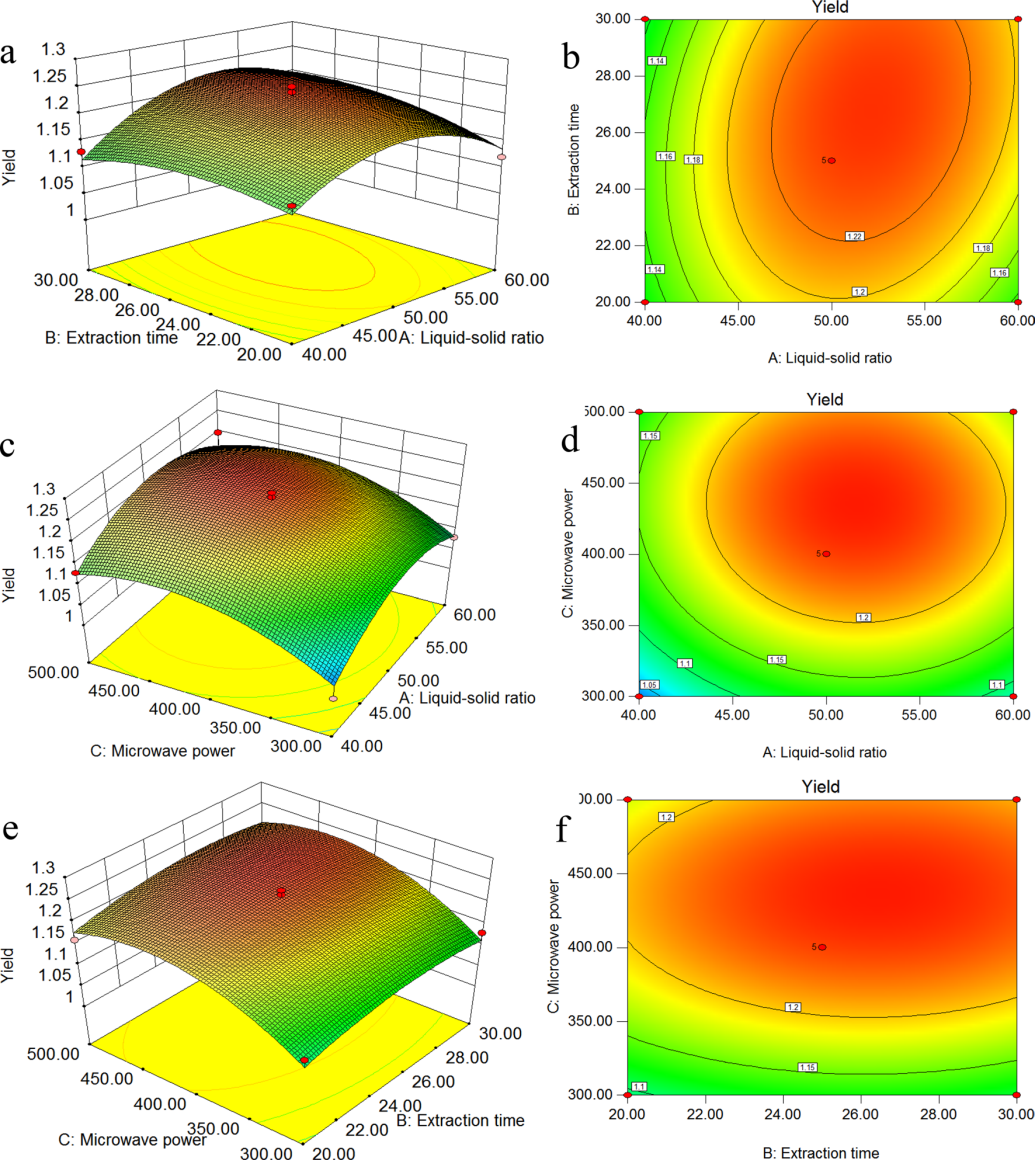
Response surface (**a**,**c**,**e**) and contours (**b**,**d**,**f**) of the effects of the liquid–solid ratio, extraction time and microwave power.

Design Expert 8.0 software was utilized to perform quadratic multiple regression fitting of the data in Table 4. The response surface results of the quadratic regression equation are shown in Fig. 3. As shown in Fig. 3a, as the liquid–solid ratio increased, the yield appears to increase and then decrease in certain microwave power conditions. As shown in Fig. 3e, in certain liquid–solid ratio conditions, with an increase in the microwave power, the yield also appears to increase and then decrease. In this case, A, C, A^2^, and C^2^ are significant model terms, and B, AB, AC, BC, and B^2^ are nonsignificant model terms. By the response surface analysis of the regression model, with the goal of maximizing the yield of sweet cherry flower essential oil, the optimal extraction conditions for UMAE are a liquid–solid ratio of 52.06 mL g^−1^, an extraction time of 26.83 min, and microwave power of 435.94 W. In these extraction conditions, the regression equation model predicts the yield of sweet cherry flower essential oil to be 1.24667%. Considering the feasibility of the actual operating conditions, the extraction condition parameters were revised to a liquid–solid ratio of 52 mL g^−1^, an extraction time of 27 min, and microwave power of 435 W. The actual yield of essential oils measured by 3 parallel experiments is 1.23%, which is near the predicted value and indicates that the regression equation model can better simulate and predict the yield of sweet cherry flower essential oils.

### Identification of VOCs of sweet cherry flower essential oil

**Figure 4:**
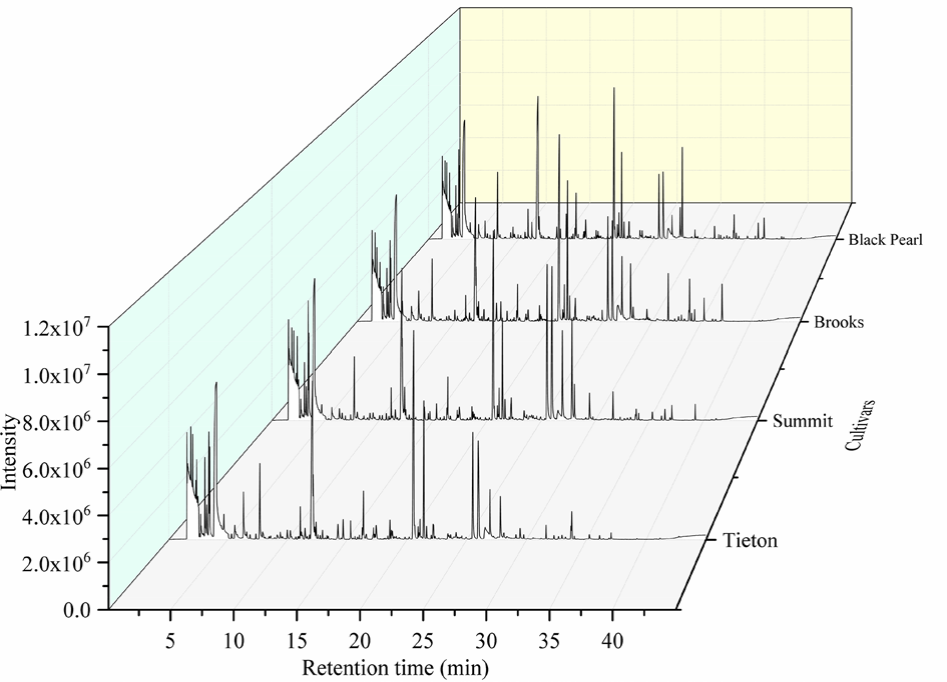
Total ion chromatogram of sweet cherry flower essential oils.

The extraction of VOCs from samples is a key link in the analysis of aromatic components. In the past, solvent extraction was employed to extract aromatic compounds, but due to its defects, this method could not completely extract VOCs from the samples, which affects the accuracy of the analysis results^35–37^. In this study, the HS-SPME was utilized to enrich the volatile and semivolatile components in the sample, combined with GC–MS technology to analyze, identify, and compare the aroma components of sweet cherry flower essential oil. This method offers high sensitivity and simple operation and is convenient and fast. The total ion chromatogram of the sweet cherry flower essential oils by HS-SPME/GC–MS separation analysis is shown in Fig. 4 The total ion chromatogram in four cultivars is similar, but the peak area with the same retention time is different. In this study, a total of 155 VOCs (Table 6) of 11 different chemical groups were separated and identified in the Brooks, Black Pearl, Tieton and Summit sweet cherry cultivars. Sweet cherry flower essential oils from different cultivars have different numbers and relative contents of VOCs.

**Table 6:** Volatile organic compounds identified in four sweet cherry cultivar flower essential oils by HS-SPME/GC–MS.

Peak no	Compound	Molecular formula	RT/min	Relative content/%			
				Brooks	Black pearl	Tieton	Summit
1	Acetaldehyde	C_2_H_4_O	1.94	5.68	5.57	6.13	4.47
2	Dimethyl sulfide	C_2_H_6_S	2.11	5.56	5.54	5.78	5.92
3	Trimethylene oxide	C_3_H_6_O	2.29	–	1.09	–	–
4	Octane	C_8_H_18_	2.36	1.59	1.67	1.71	1.79
5	Propanal, 2-methyl-	C_4_H_8_O	2.43	2.40	2.55	2.72	3.01
6	Ethyl ether	C_4_H_10_O	2.52	–	–	0.82	–
7	2-Propenal	C_3_H_4_O	2.68	–	0.13	–	0.13
8	Cyclotrisiloxane, hexamethyl-	C_6_H_18_O_3_Si_3_	2.76	0.81	0.59	0.48	0.48
9	Butanal	C_4_H_8_O	2.97	0.14	0.15	0.10	0.12
10	CH_3_C(O)CH_2_CH_2_OH	C_4_H_8_O_2_	3.11	1.55	1.40	1.78	1.45
11	Furan, 3-methyl-	C_5_H_6_O	3.19	–	0.22	0.19	0.18
12	Butanal, 2-methyl-	C_5_H_10_O	3.45	2.21	2.73	2.55	3.38
13	Butanal, 3-methyl-	C_5_H_10_O	3.51	2.17	2.66	2.47	2.96
14	Ethanol	C_2_H_6_O	4.07	13.67	13.68	20.67	15.42
15	Cyclohexene, 1-methyl-	C_6_H_9_CH_3_	4.16	–	–	–	1.96
16	Heptane, 2,2,4,6,6-pentamethyl-	C_12_H_26_	4.35	–	0.71	0.70	0.56
17	2-Ethylacrolein	C_5_H_8_O	4.43	–	–	–	0.31
18	Propanoic acid, 2-methyl-, ethyl ester	C_6_H_12_O_2_	4.48	0.88	0.85	0.56	0.81
19	3-Pentanone	C_5_H_10_O	4.70	–	1.83	0.72	–
20	Decane	C_10_H_22_	5.34	0.11	0.04	0.17	0.04
21	trans-4-Dimethylamino-4′-methoxychalcone	C_18_H_19_NO_2_	5.68	0.45	–	–	–
22	(1R)-2,6,6-Trimethylbicyclo[3.1.1]hept-2-ene	C_10_H_16_	5.80	0.55	–	0.21	0.37
23	2-Butanol	C_4_H_10_O	6.22	0.09	–	–	0.07
24	2-Butenal, (*E*)-	C_4_H_6_O	6.34	1.49	0.89	1.90	0.58
25	1-Propanol	C_3_H_8_O	6.60	0.41	0.30	0.31	0.32
26	Butanoic acid, 2-methyl-, ethyl ester	C_7_H_14_O_2_	6.89	0.14	0.14	0.12	0.14
27	Butanoic acid, 3-methyl-, ethyl ester	C_7_H_14_O_2_	7.37	0.14	0.28	0.14	0.15
28	Hexanal	C_6_H_12_O	7.71	2.36	2.78	2.49	2.25
29	2-Butenal, 2-methyl-	C_5_H_8_O	7.99	0.20	0.20	0.21	0.45
30	.beta.-Pinene	C_10_H_16_	8.14	–	–	–	0.08
31	Tetrasiloxane, decamethyl-	C_10_H_30_O_3_Si_4_	8.48	–	–	–	0.07
32	1-Propanol, 2-methyl-	C_4_H_10_O	8.61	0.11	0.09	0.12	0.24
33	1-Butanol, 3-methyl-, acetate	C_7_H_14_O_2_	8.94	0.03	0.04	0.04	0.14
34	2-Pentenal, (*E*)-	C_5_H_8_O	9.17	–	0.32	0.11	0.36
35	o-Xylene	C_8_H_10_	9.20	–	–	0.12	–
36	3-Hexenal	C_6_H_10_O	9.42	–	0.62	0.32	–
37	.alpha.-Phellandrene	C_10_H_16_	9.88	0.03	–	–	–
38	.beta.-Myrcene	C_10_H_16_	10.01	0.46	0.19	0.32	0.24
39	2-Butenoic acid, ethyl ester, (*E*)-	C_6_H_10_O_2_	10.27	–	–	0.36	–
40	1-Butanol	C_4_H_10_O	10.37	0.10	0.09	0.11	–
41	1-Penten-3-ol	C_5_H_10_O	10.74	–	0.29	–	–
42	Heptanal	C_7_H_14_O	10.90	0.09	0.10	0.08	0.15
43	d-Limonene	C_10_H_16_	10.98	0.15	0.06	0.12	–
44	Limonene	C_10_H_16_	11.04	–	–	–	0.07
45	Cyclopentasiloxane, decamethyl-	C_10_H_30_O_5_Si_5_	11.10	0.64	0.94	0.72	0.76
46	Dodecane	C_12_H_26_	11.21	0.25	–	0.28	0.18
47	.beta.-Phellandrene	C_10_H_16_	11.27	0.12	–	0.15	–
48	(2R,5R)-2-Methyl-5-(prop-1-en-2-yl)-2-vinyltetrahydrofuran	C_10_H_16_O	11.67	0.17	0.15	0.12	0.13
49	(*E*)-2-Hexenal	C_6_H_10_O	12.12	8.51	16.50	10.10	10.78
50	1-Butanol, 3-methyl-	C_5_H_12_O	12.17	2.23	–	2.32	2.53
51	Furan, 2-pentyl-	C_9_H_14_O	12.32	0.57	1.00	0.43	1.16
52	trans-.beta.-Ocimene	C_10_H_16_	12.42	0.68	0.30	0.55	0.41
53	Hexanoic acid, ethyl ester	C_8_H_16_O_2_	12.58	0.09	0.18	0.17	0.12
54	(2R,5S)-2-Methyl-5-(prop-1-en-2-yl)-2-vinyltetrahydrofuran	C_10_H_16_O	12.77	0.17	0.12	0.13	0.22
55	.beta.-Ocimene	C_10_H_16_	12.96	0.52	0.08	0.38	0.13
56	1,3,5,7-Cyclooctatetraene	C_8_H_8_	13.08	0.03	–	0.03	0.04
57	3-Octanone	C_8_H_16_O	13.16	–	0.04	0.05	–
58	3-Buten-1-ol, 3-methyl-	C_5_H_10_O	13.27	–	–	–	0.06
59	1-Pentanol	C_5_H_12_O	13.39	–	–	–	0.12
60	Benzene, 1-methyl-3-(1-methylethyl)-	C_10_H_14_	13.42	–	0.09	0.13	–
61	Acetic acid, hexyl ester	C_8_H_16_O_2_	13.75	–	0.03	0.03	–
62	2-Hexanol	C_6_H_14_O	14.20	–	–	–	0.61
63	Tridecane	C_13_H_28_	14.50	–	–	0.09	–
64	cis-2-(2-Pentenyl)furan	C_9_H_12_O_2_	14.53	0.08	0.15	–	0.22
65	(*E*)-4,8-Dimethylnona-1,3,7-triene	C_11_H_18_	14.68	0.68	0.42	0.65	0.28
66	2-Heptenal, (*E*)-	C_7_H_12_O	15.21	–	–	0.03	0.03
67	3-Octanone, 2-methyl-	C_9_H_18_O	15.30	0.35	0.89	–	–
68	2-Penten-1-ol, (*Z*)-	C_5_H_10_O	15.40	–	0.06	–	–
69	2,3-Octanedione	C_8_H_14_O_2_	15.47	–	–	–	0.05
70	5-Hepten-2-one, 6-methyl-	C_8_H_14_O	15.66	0.08	0.06	–	0.09
71	1,6-Dioxaspiro[4.4]nonane, 2-ethyl-	C_9_H_16_O_2_	16.16	0.02	–	0.02	–
72	1-Hexanol	C_6_H_14_O	16.36	1.30	1.83	1.51	1.53
73	2,4,6-Octatriene, 2,6-dimethyl-, (*E*,*Z*)-	C_10_H_16_	16.56	0.20	0.05	0.08	–
74	3-Hexen-1-ol, (*E*)-	C_6_H_12_O	16.61	–	0.20	0.16	0.15
75	3-Hexen-1-ol	C_6_H_12_O	17.16	–	–	–	0.06
76	Nonanal	C_9_H_18_O	17.21	0.35	0.37	0.47	0.31
77	2,4-Hexadienal, (*E*,*E*)-	C_6_H_8_O	17.31	0.15	0.32	0.21	0.24
78	Furan, 2-ethyl-	C_6_H_8_O	17.43	–	0.93	0.64	0.66
79	1,3-Hexadiene, 3-ethyl-2-methyl-	C_9_H_16_	17.77	0.02	0.04	0.03	0.05
80	Furan, 3-(4-methyl-3-pentenyl)-	C_10_H_14_O	17.89	0.02	–	–	0.02
81	Sulfurous acid, cyclohexylmethyl tridecyl ester	C_23_H_46_O_3_S	18.09	–	–	–	0.02
82	2-Octenal, (*E*)-	C_8_H_14_O	18.17	0.05	–	–	0.07
83	Benzene, 1-methyl-4-(1-methylethenyl)-	C_10_H_12_	18.32	0.06	0.03	0.05	0.02
84	Octanoic acid, ethyl ester	C_10_H_20_O_2_	18.41	0.09	0.10	0.11	0.09
85	2,6,10-Trimethyltridecane	C_16_H_34_	18.59	0.46	0.28	0.52	0.36
86	Methional	C_4_H_8_OS	18.80	0.18	0.32	0.28	0.23
87	Acetic acid	C_2_H_4_O_2_	18.93	0.10	0.18	0.13	0.17
88	3-Furaldehyde	C_5_H_4_O_2_	19.04	0.03	–	–	–
89	Cyclotetrasiloxane, octamethyl-	C_8_H_24_O_4_Si_4_	19.31	–	0.04	–	–
90	Ethyl 2-(5-methyl-5-vinyltetrahydrofuran-2-yl)propan-2-yl carbonate	C_13_H_22_O_4_	19.44	–	–	0.02	–
91	2,4-Heptadienal, (*E*,*E*)-	C_7_H_10_O	19.84	0.08	0.10	0.09	0.13
92	2-Ethyl-1-hexanol		19.98	–	0.05	0.03	0.05
93	Pentadecane	C_15_H_32_	20.14	–	0.03	0.01	–
94	trisiloxane, 1,1,1,5,5,5-hexamethyl-3-[(trimethylsilyl)oxy]-	C_9_H_27_O_3_Si_4_	20.18	0.08	0.11	–	–
95	Benzaldehyde	C_7_H_6_O	20.57	13.04	8.49	10.05	10.03
96	2-Nonenal, (*E*)-	C_9_H_16_O	20.94	0.34	0.45	0.31	0.41
97	Lilac aldehyde B	C_10_H_16_O_2_	21.10	1.18	0.90	0.58	1.01
98	Propanoic acid	C_3_H_6_O_2_	21.21	0.08	0.10	0.12	–
99	Linalool	C_10_H_18_O	21.41	5.50	3.15	4.17	3.43
100	Dimethyl sulfoxide	C_2_H_6_OS	22.04	0.12	0.10	0.12	0.10
101	2,6-Nonadienal, (*E*,*Z*)-	C_9_H_14_O	22.19	–	0.49	0.38	–
102	Lilac aldehyde C	C_10_H_16_O_2_	22.22	0.99	–	0.34	0.97
103	3-Cyclohexene-1-acetaldehyde, .alpha.,4-dimethyl-	C_10_H_16_O	22.93	0.05	–	–	–
104	1-Cyclohexene-1-carboxaldehyde, 2,6,6-trimethyl-	C_10_H_16_O	22.99	–	0.05	0.02	0.04
105	Butyrolactone	C_4_H_6_O_2_	23.07	–	–	0.03	0.02
106	Ethanol, 2-(2-ethoxyethoxy)-	C_6_H_14_O_3_	23.15	0.03	0.03	–	–
107	Benzeneacetaldehyde	C_8_H_8_O	23.43	–	–	0.27	0.43
108	Decanoic acid, ethyl ester	C_12_H_24_O_2_	23.53	0.14	0.20	0.17	0.17
109	Silanediol, dimethyl-	C_2_H_8_O_2_Si	23.92	0.38	–	0.21	0.39
110	1-Nonanol	C_9_H_20_O	24.11	0.25	–	0.35	0.35
111	Benzaldehyde, 2-hydroxy-	C_7_H_6_O_2_	24.26	0.12	0.11	0.08	0.17
112	Butanoic acid, 3-methyl-	C_5_H_10_O_2_	24.42	–	0.16	–	–
113	3-Pyridinecarboxaldehyde	C_6_H_5_NO	24.94	0.47	0.18	0.14	0.18
114	1-Propanol, 3-(methylthio)-	C_4_H_10_OS	25.33	0.04	0.06	0.04	0.08
115	Lilac alcohol B	C_10_H_18_O_2_	25.97	4.91	–	–	–
116	Benzene, 1-methoxy-2-(methoxymethyl)-	C_9_H_12_O_2_	26.18	0.06	–	0.03	–
117	Lilac alcohol formate B	C_11_H_18_O_3_	26.33	–	–	–	0.06
118	Oxime-, methoxy-phenyl-_	C_8_H_9_NO_2_	26.55	–	2.18	1.60	1.55
119	Lilac alcohol C	C_10_H_18_O_2_	26.95	3.09	1.28	2.00	3.75
120	Dodecanoic acid, methyl ester	C_13_H_26_O_2_	27.24	–	–	–	0.17
121	Tris(tert-butyldimethylsilyloxy)arsane	C_18_H_45_AsO_3_Si_3_	27.69	–	0.19	–	–
122	3,5-Dimethoxytoluene	C_9_H_12_O_2_	28.08	0.70	3.49	0.14	1.21
123	Dodecanoic acid, ethyl ester	C_14_H_28_O_2_	28.13	–	–	0.15	–
124	Hexanoic acid	C_6_H_12_O_2_	28.38	0.19	0.07	0.10	–
125	Benzyl alcohol	C_7_H_8_O	28.73	–	–	–	0.03
126	Phenylethyl alcohol	C_8_H_10_O	29.47	0.51	0.35	0.36	1.01
127	3-Buten-2-one, 4-(2,6,6-trimethyl-1-cyclohexen-1-yl)-	C_13_H_20_O	30.05	–	0.03	–	0.02
128	2-Cyclopenten-1-one, 3-methyl-2-(2-pentenyl)-, (*Z*)-	C_11_H_16_O	30.14	–	–	0.02	–
129	Benzaldehyde, 2-methoxy-	C_8_H_8_O_2_	30.40	–	0.01	–	–
130	Phenol	C_6_H_6_O	31.29	–	–	0.02	0.03
131	Methyleugenol	C_11_H_14_O_2_	31.48	–	0.04	–	0.05
132	Benzaldehyde, 4-methoxy-	C_8_H_8_O_2_	31.64	1.50	0.45	0.42	0.86
133	Benzene, 1,2,3-trimethoxy-5-methyl-	C_10_H_14_O_3_	32.08	0.02	0.14	–	0.04
134	Tetradecanoic acid, ethyl ester	C_16_H_32_O_2_	32.29	0.10	0.13	0.13	0.13
135	2-Propenoic acid, 3-phenyl-, methyl ester, (*E*)-	C_10_H_10_O_2_	32.68	0.20	0.10	0.10	0.06
136	Benzoic acid, 4-methoxy-, methyl ester	C_9_H_10_O_3_	32.97	0.17	0.14	0.13	0.08
137	Heneicosane	C_21_H_44_	33.27	0.10	0.10	0.06	0.11
138	2-Propenoic acid, 3-phenyl-, ethyl ester, (*E*)-	C_11_H_12_O_2_	33.72	0.29	0.26	0.32	0.15
139	Benzoic acid, 4-methoxy-, ethyl ester	C_10_H_12_O_3_	33.79	1.29	0.81	0.77	0.33
140	2,4,6-Cycloheptatrien-1-one, 2-amino-	C_7_H_7_NO	34.31	0.40	0.12	0.02	0.02
141	1-Hexadecen-3-ol, 3,5,11,15-tetramethyl-	C_20_H_40_O	35.26	–	–	0.16	–
142	Hexadecanoic acid, methyl ester	C_17_H_34_O_2_	35.42	–	0.02	–	0.03
143	Hexadecanoic acid, ethyl ester	C_18_H_36_O_2_	36.11	0.08	0.11	0.12	0.14
144	3,5-Dimethoxybenzaldehyde	C_9_H_10_O_3_	36.44	0.09	0.56	0.03	0.35
145	Eicosane	C_20_H_42_	36.90	0.05	0.09	0.06	0.12
146	2,7-Octadiene-1,6-diol, 2,6-dimethyl-	C_10_H_18_O_2_	37.07	1.44	0.87	0.24	0.57
147	Benzoic acid, 3,5-dimethoxy-, methyl ester	C_10_H_12_O_4_	38.48	–	0.03	–	–
148	Coumarin	C_9_H_6_O_2_	39.24	–	0.09	0.04	0.54
149	9,12-Octadecadienoic acid, ethyl ester	C_20_H_36_O_2_	40.73	–	0.02	0.02	0.02
150	Phthalic acid, isobutyl trans-hex-3-enyl ester	C_18_H_24_O_4_	40.86	0.03	0.02	0.02	–
151	Vanillin	C_8_H_8_O_3_	41.16	–	0.07	–	0.06
152	1,4,7,10,13,16-Hexaoxacyclooctadecane	C_12_H_24_O_6_	42.47	0.01	–	–	–
153	Dibutyl phthalate	C_16_H_22_O_4_	42.74	0.07	–	–	–
154	2-[2-[2-[2-[2-[2-[2-(2-Methoxyethoxy)ethoxy]ethoxy]ethoxy]ethoxy]ethoxy]ethoxy]ethanol	C_17_H_36_O_9_	43.47	–	–	0.14	–
155	2-[2-[2-[2-[2-[2-(2-Methoxyethoxy)ethoxy]ethoxy]ethoxy]ethoxy]ethoxy]ethanol	C_15_H_32_O_8_	43.83	–	–	0.10	–

*RT* retention time.

The VOCs possessed by plant flowers are secondary metabolites, which have a vital role in the pollination process of plants. Lilac alcohol, dimethyl sulfide, acetaldehyde, 3-methyl-1-butanol, 3-methyl butanal, (*E*)-2-hexenal, benzaldehyde, etc. in the VOCs of sweet cherry flowers can be employed in natural floral flavors. Among them, lilac alcohol has a lilac fragrance. Dimethyl sulfide is one of the key odorants in the production of corn, tomato, potatoes, dairy product, and pineapple^38^. In addition, 3-methylbutanal is mainly employed to formulate various fruit flavors. Ethanol, linalool, 4-methoxy-benzaldehyde and lilac aldehyde have been shown to have certain pharmacological effects^39^. A large amount of ethanol is observed in the flowers of sweet cherry during the full bloom period, which is caused by the change in carbon metabolism during the glycolysis of flowers. As the flower matures, respiration increases, and pyruvate, which is the intermediate product of glycolysis, enters the tricarboxylic acid cycle. The remaining part is converted to ethanol, which causes an accumulation of ethanol. The large amount of ethanol not only harms the flowers but also weakens the tolerance and disease resistance of the plant and increases the occurrence of decay. Linalool has many pharmacological activities, such as analgesic, sedative hypnosis, antianxiety and antitumor activities^40,41^. Moreover, 4-methoxy-benzaldehyde is employed in medicine as an intermediate for antihistamine drugs, such as the antibiotic hydroxyaminobenzyl penicillin. The types, contents and proportions of VOCs of sweet cherry flowers have a strong influence in attracting pollinators to forage (e.g., the noctuid moth *Hadena bicruris* relies on lilac aldehyde to find its host plant)^39^.

### Classification analysis of VOCs

To identify the main aroma compounds in sweet cherry flower essential oil, the differences in the VOCs of the essential oils of the flours of the four sweet cherry cultivars were compared. The VOCs were grouped according to their chemical families as alcohols, aldehydes, esters, ether, furan, alkane, olefin, terpenes, ketones, organic acids, and other VOCs.

**Figure 5:**
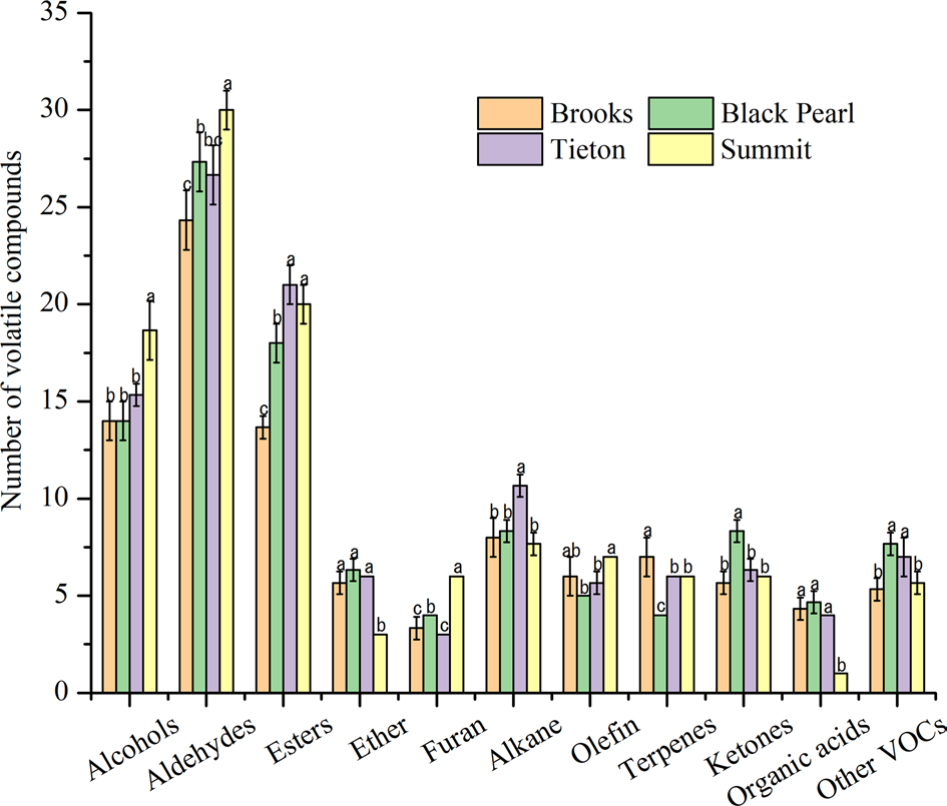
Number of volatile organic compounds of sweet cherry flower essential oils. The vertical bars represent the standard deviation of the means (n = 3). Different letters (for the same compound) indicate significant differences (p < 0.05).

As shown in Fig. 5, in the Brooks, Black Pearl, Tieton, and Summit cultivars, 97 compounds, 107 compounds, 112 compounds and 111 compounds, respectively, were detected. Among the 155 VOCs, ethanol, linalool, dimethyl sulfide, acetaldehyde, (*E*)-2-hexenal, and benzaldehyde are the main compounds that comprise the aroma of the sweet cherry flower essential oil from the four cultivars.

**Figure 6:**
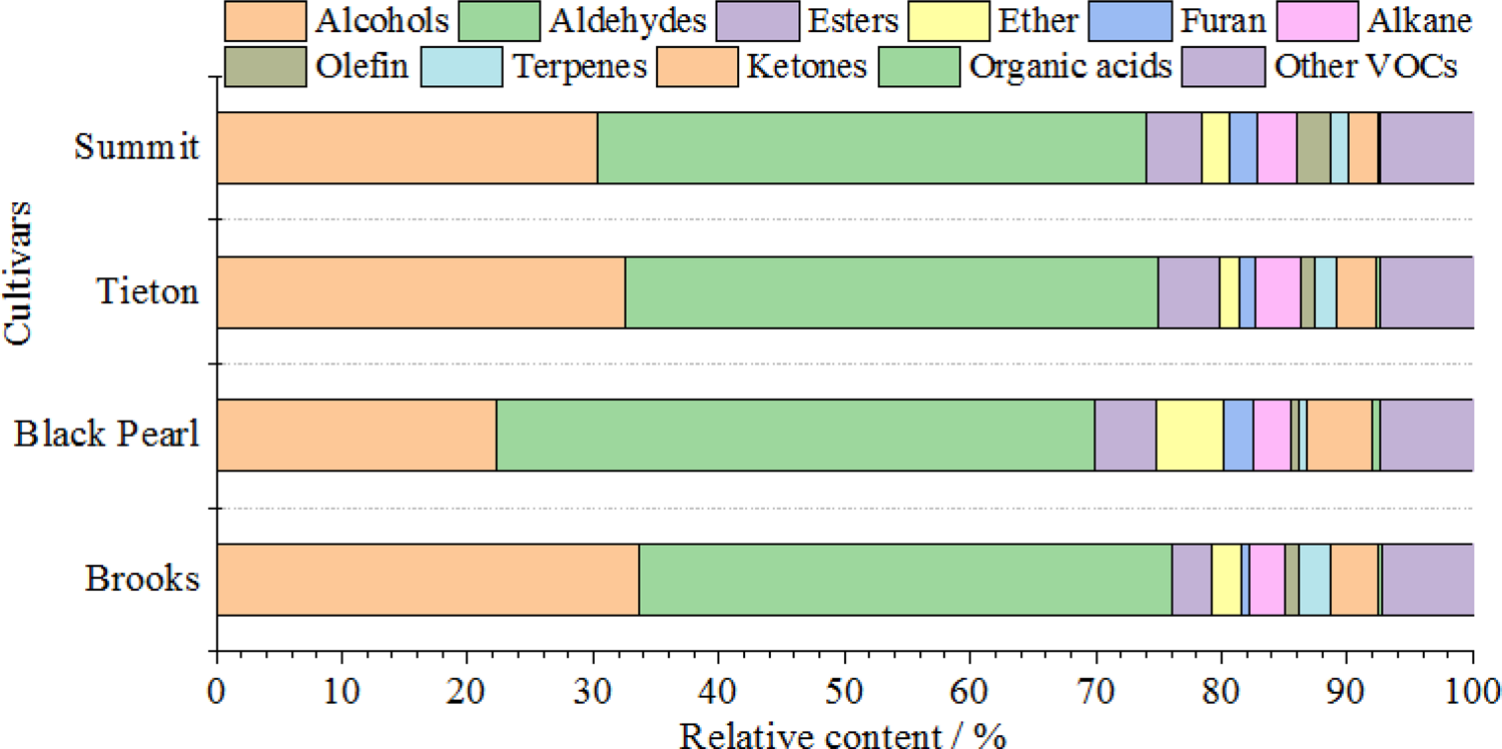
Relative contents of volatile organic compounds of sweet cherry flower essential oils.

It can be seen from Fig. 6 that the aldehyde content is the largest in the VOCs, followed by alcohols. These two substances are the main sources of sweet cherry flower aroma. Benzaldehyde was detected in four sweet cherry cultivar flowers and hyacinth, citronella, cinnamon, iris and rose. Moreover, (*E*)-2-hexenal has a fresh green leaf fragrance and can be used as a blending fragrance for essential oils and various floral fragrances^42^. Acetaldehyde is also present in the aroma of four sweet cherry cultivar flowers and naturally exists in round pomelo, pear, apple, raspberry, strawberry, pineapple, coffee, and orange juice^43^. After dilution, acetaldehyde has a fruity, coffee, wine, green fragrance. Benzaldehyde and benzeneacetaldehyde are important aldehyde flavor ingredients^44^, and benzaldehyde has the aroma of bitter almond, cherry and nut. Benzaldehyde, which is a common component of plant volatiles^45^, attracts many pest species. A recent study has indicated that benzaldehyde can be recognized by adult *A. lucorum* and can affect its behavior^46,47^. Benzaldehyde is produced by enzymolysis of amygdalin in flowers, and the importance of this substance has been emphasized in previous studies^48^. In this study, the concentrations of benzaldehyde and its derivative benzyl alcohol in the essential oil of the four sweet cherry cultivar flowers are relatively high, which shows the best fragrance of the flowers. This result confirms the importance of benzaldehyde to the sweet cherry flower aroma. Hexanal is a fatty acid that is produced under the catalytic action of lipoxygenase (LOX)^49^. Hexanal has the fragrance of grass and can increase the perceived intensity of fruit aroma. Linalool and phenylethyl alcohol are important alcohol flavor ingredients, among which linalool is extensively applied in cosmetic flavors and food fruit flavors with antibacterial and antiviral effects. Phenylethyl alcohol is one of the two main aroma components of rose essential oil^50^. The fresh, sweet smell of phenylethyl has calming and soothing effects and anti-inflammatory and antibacterial effects. Lilac alcohol isomers (lilac alcohol B and lilac alcohol C) and lilac aldehyde isomers (lilac aldehyde B and lilac aldehyde C) in sweet cherry flowers are the characteristic aroma components. Most esters can impart plant fruit fragrance. 4-Methoxybenzoic acid ethyl ester is the main ester component of sweet cherry flowers, with aromas similar to fruits and anise. Among the olefins, beta-pinene, myrcene, trans-beta-ocimene, and β-ocimene are the main components, and beta-pinene is only detected in the Summit cultivar^51^. Ocimene has a certain role in the prevention and treatment of cancer. An antidepressant experiment in mice has also shown that ocimene can effectively reduce depression traits in mice^52^. Ethers and ketones are relatively rare in flowers. Ketones are also typical aromatic components, and 4-hydroxy-2-butanone is typical of sweet cherry flowers. These VOCs provide the unique aromatic quality of sweet cherry flowers, which indicates that sweet cherry flowers are an important natural spice raw material, which has important scientific value and excellent development prospects.

## Conclusions

Ultrasound- and microwave-assisted extraction is an effective method that is suitable for the extraction of essential oil from sweet cherry flowers. Response surface methodology, as part of the experimental design and optimization, showed that the liquid–solid ratio and microwave power had a notable influence on the extraction yield. HS-SPME/GC–MS is an accurate, fast and effective method for determining the aromatic components of sweet cherry flower essential oil. The analysis results concluded that the detected VOCs in the Brooks, Black Pearl, Tieton and Summit sweet cherry cultivars were similar. However, significant variations in the contributions of these compounds to each cultivar were observed. Regardless of the cultivar, the most abundant alcohols and aldehyde compounds were ethanol and benzaldehyde, respectively. The principal volatiles were ethanol, linalool, lilac alcohol, acetaldehyde, (*E*)-2-hexenal, benzaldehyde and dimethyl sulfide, and their concentrations were highly dependent on each cultivar. These VOCs are the main sources of the aroma of sweet cherry flowers. Ethanol, linalool, 4-methoxy-benzaldehyde and lilac aldehyde have various biological activities. The research results provide a basis for the health benefits of sweet cherry flowers.
